# Development of a multivariable improvement measure for gout

**DOI:** 10.1186/s13075-020-02254-4

**Published:** 2020-06-29

**Authors:** Naomi Schlesinger, N. Lawrence Edwards, Anthony E. Yeo, Peter E. Lipsky

**Affiliations:** 1 Division of Rheumatology, Department of Medicine, Rutgers Robert Wood Johnson Medical School, New Brunswick, NJ USA; 2 Department of Medicine, University of Florida, Gainesville, FL USA; 3 Yeo Analytics, LLC, Jersey City, NJ USA; 4 AMPEL BioSolutions, LLC, Charlottesville, VA USA

**Keywords:** Gout, Disease activity, Pegloticase

## Abstract

**Background:**

Gout is a heterogeneous inflammatory disease with numerous clinical manifestations. A composite means to assess the impact of therapy on numerous aspects of gout could be useful.

**Methods:**

Results from patients treated with pegloticase or placebo in two randomized clinical trials and their open-label extension were assessed using a novel evidence-based Gout Multivariable Improvement Measure (GMIM) derived from previously reported criteria for remission and complete response. Improvement was defined as serum urate (sU) < 6 mg/dL and absence of flares during the preceding 3 months plus 20, 50, and 70% improvement in tophus size, patient global assessment, pain, and swollen and tender joints.

**Results:**

Patients treated with pegloticase manifested a significantly greater GMIM20, 50, and 70 response vs those treated with placebo (GMIM20 at 6 months 37.1% vs 0%, respectively). Higher response rates were significantly more frequent in subjects with persistent urate lowering (GMIM 58.1% at 6 months) in response to pegloticase versus those with only transient urate lowering (GMIM 7.1% at 6 months). However, when the requirement for a decrease in sU to < 6 mg/dL was omitted, a substantial percentage of subjects with transient urate lowering met the GMIM clinical criteria. A sensitivity analysis indicated that gout flares contributed minimally to the model. The response measured by GMIM persisted into the open-level extension for as long as 2 years. Finally, subjects who received placebo in the randomized control trials, but pegloticase in the open-label extension, manifested GMIM responses comparable to that noted with pegloticase-treated subjects in the randomized controlled trials.

**Conclusions:**

GMIM captures changes in disease activity in response to treatment with pegloticase and may serve as an evidence-based tool for assessment of responses to other urate-lowering therapies in gout patients.

## Key messages

What is already known about this subject?

- A Gout Activity Score (GAS) has been proposed, but its utility in clinical trials has not been documented.
- Tools to assess response to gout treatment are needed.

What does this study add?

- A new Gout Multivariable Improvement Measure (GMIM) was developed based on evidence from previous clinical trials.
- The GMIM captures both biochemical and clinical information and is sensitive to change.

How might this impact on clinical practice or future developments?

- The GMIM may provide a means to assess clinical benefit of urate-lowering therapy in clinical trials and medical care.

## Background

Inflammatory rheumatic diseases are generally multifaceted disorders, and the complex pathology underlying these conditions makes it difficult to assess patient status and the efficacy of therapy with a single representative outcome measure [1]. The lack of a single gold standard for patient monitoring has prompted the development of composite measures for many rheumatic diseases [2], and there are well-established indices for rheumatoid arthritis [3, 4], psoriatic arthritis [5–7], systemic lupus erythematosus [8–10], ankylosing spondylitis [11], osteoarthritis [12, 13], and fibromyalgia [14].

Gout is also a multifactorial inflammatory disease. Even though the cause of gout, namely hyperuricemia, is known, patients experience a wide range of symptoms, including severe pain, acute and persistent inflammatory arthritis, tophi, and disability associated with both flares and chronic disease [15, 16]. Moreover, the spectrum of disease changes with time [17, 18]. It has been recommended that multiple domains should be evaluated when assessing effects of treatment for gout [19, 20]. Although assessment of urate-lowering therapy (ULT) for this disease has focused primarily on the ability to lower serum urate (sU) and decrease in the frequency of flares [21–25], some trials have included additional endpoints to address effects of treatment on pain, arthritis, and disability [26, 27].

The overall goal of ULT is the dissolution of all urate deposits and the prevention of new deposits from occurring. Although it is assumed that this occurs when the sU level is maintained at a level < 6 mg/dL, there is no direct means other than imaging modalities to assess this. Therefore, there is the general recognition that assessment of ULT and other treatments for gout could be facilitated by endpoints that more closely reflected the multidimensional effects of urate deposition. This has prompted multiple groups to propose composite measures aimed at this goal. Over a decade ago, an Outcome Measures in Rheumatology (OMERACT) special interest group proposed core domains for interventional studies in chronic gout that included sU, gout flare recurrence, tophus regression, joint damage imaging, health-related quality of life, musculoskeletal function, patient global assessment (PGA), ability to participate in usual activities, and safety and tolerability [28]. Since that time, there have been multiple discussions regarding the domains most important for inclusion in a composite measure for use in clinical trials of advanced gout [29]. These measures are generally aimed at determining the patient’s status at a given point in time rather than providing a change score that captures response to treatment over time. Despite very high interest and intensive deliberation, development and testing of composite measures for either acute or advanced gout has been very limited. A composite index for evaluation of treatments for acute gout has been developed [30], and a composite Gout Activity Score (GAS) has been shown to be sensitive to change and to have predictive validity with a correlation to the domains of the Gout Impact Scale (GIS) [31, 32]; however, these composite measures have not been tested in trials of ULT.

The present study used results from two identical randomized controlled trials (RCTs) of pegloticase (NCT00325195, NCT01356498) for development of a composite measure for capturing responses to gout treatment. The construction of the Gout Multivariable Improvement Measure (GMIM) involved evaluation of criteria proposed to define remission in gout and included sU, frequency of flares, tophus reduction, pain evaluation, and PGA [33]. Previous evaluation of patient responses added tender and swollen joints to the list of patient features incorporated [34]. GMIM therefore comprised sU, flares, tophi, PGA, pain, and swollen and tender joint counts (SJC, TJC). Improvement was defined as sU < 6 mg/dL and absence of flares during the preceding 3 months plus 20%, 50%, or 70% improvement in ≥ 3 of the other 5 clinical evaluations.

The aim of this study was to use GMIM to assess the degree of response in patients with chronic refractory gout treated with pegloticase and to validate the composite response measure by comparing its ability to discriminate those with persistent urate lowering from pegloticase treatment with those with transient urate lowering and also those receiving placebo. GMIM was also tested in a group of patients who received placebo in the RCTs and began pegloticase in the open-label extension to evaluate the composite measure in a situation not used to develop the tool. Although based on data from a subset of subjects with advanced gout, the tool may have utility in other settings.

## Methods

### Design of pegloticase clinical trials

The design of the two identical RCTs of pegloticase and their open-label extension (OLE) that provided the data analyzed in this study have been described in detail previously [26, 35] and are only briefly summarized here. Both studies received institutional review board approval for each site, and written informed consent and Health Insurance Portability and Accountability Act assurances were completed for each participant prior to enrollment. These trials included adults with chronic refractory gout with one or more of the following: sU ≥ 8.0 mg/dL and ≥ 3 self-reported gout flares during the previous 18 months; ≥ 1 tophus; chronic gouty arthritis; and failure to respond to the maximum medically appropriate allopurinol dose, as determined by the treating physician, or a contraindication to this drug. Patients were randomized to 6 months of intravenous infusions of either pegloticase 8 mg every 2 weeks (q2w), every 4 weeks (q4w), or placebo [26]. The primary endpoint for the RCTs was reduction of sU (sU < 6.0 mg/dL) ≥ 80% of the time during month 3 (extending from the week-9 infusion to just before the week-13 infusion) and month 6 (extending from the week-21 infusion to the week-25 final study visit). Any patient not achieving this goal or who did not complete the trial was classified as a nonresponder. Patients from the RCTs could continue into the OLE in which they were given the choice of receiving pegloticase q2w or q4w [35].

These trials had a large number of secondary endpoints which made results particularly useful for development of a single composite outcome measure, including tophus resolution; reduction in gout flares; decreases in TJC and SJC; PGA of disease activity; and patient-reported changes in pain, physical function (HAQ–Disability Index), and quality of life (36-Item Short Form Health Survey) [26]. Patients were assessed at baseline and at the week 13 and 19 visits as well as at the week 25 final visit for secondary endpoints. In addition, patients were evaluated for up to 27 months of the OLE.

### Construction of the Gout Multivariable Improvement Measure (GMIM)

A total of 85 subjects were treated with biweekly pegloticase. Overall, 42% (*n* = 36) of the patients who received pegloticase 8 mg every 2 weeks were responders (persistently lowered sU) to treatment in the RCTs. In previous work, responders were evaluated to determine whether they met combined criteria for remission defined by a previous Delphi exercise [33]. In addition, the clinical data from the responders was employed to develop criteria for a complete response. This was derived by employing a repeated measures mixed effects model with backward elimination that related clinical and laboratory changes observed throughout the trial [34]. In this exercise, numerous outcome measures were considered, but sU, PGA, numbers of tender and swollen joints, and the degree of tophus resolution controlling for repeated measures were found to contribute the most information to the complete response model [34]. Based upon the previous criteria for remission and complete response, the final set of criteria employed in GMIM were identified and included sU < 6 mg/dL, PGA scores, visual analog scale (VAS) pain levels, TJC and SJC, the number of flare episodes, and the degree of tophus area resolution. Improvement was defined as sU < 6 mg/dL and the absence of flares during the preceding 3 months plus 20%, 50%, or 70% improvement in ≥ 3 of the other 5 clinical criteria. Because improvement in sU and a decrease in flare frequency were uniformly used in clinical trials and were felt to be mandatory components of response, these were required along with a variety of clinical features that might vary in different patients. Since sU can vary with time, evaluation of clinical responses was also carried out with and without the requirement for achievement of sU < 6 mg/dL. Sensitivity analysis was done by removing one variable at a time from the analysis.

### Statistical analysis

All comparisons were carried out with Fisher’s exact test to determine whether there was a nonrandom association between two variables. Chi-square with correction for continuity was used when the frequency of events was small. Statistical significance was defined as *p* < 0.05. Components that contributed the most information to the GMIM model were determined by multiple linear regression with backward elimination of the least statistically significant term to model the relationship between the dependent variable, the GMIM response, and various independent variables.

All calculations were carried out with SAS version 9.4 (Cary, NC).

## Results

**Fig. 1:**
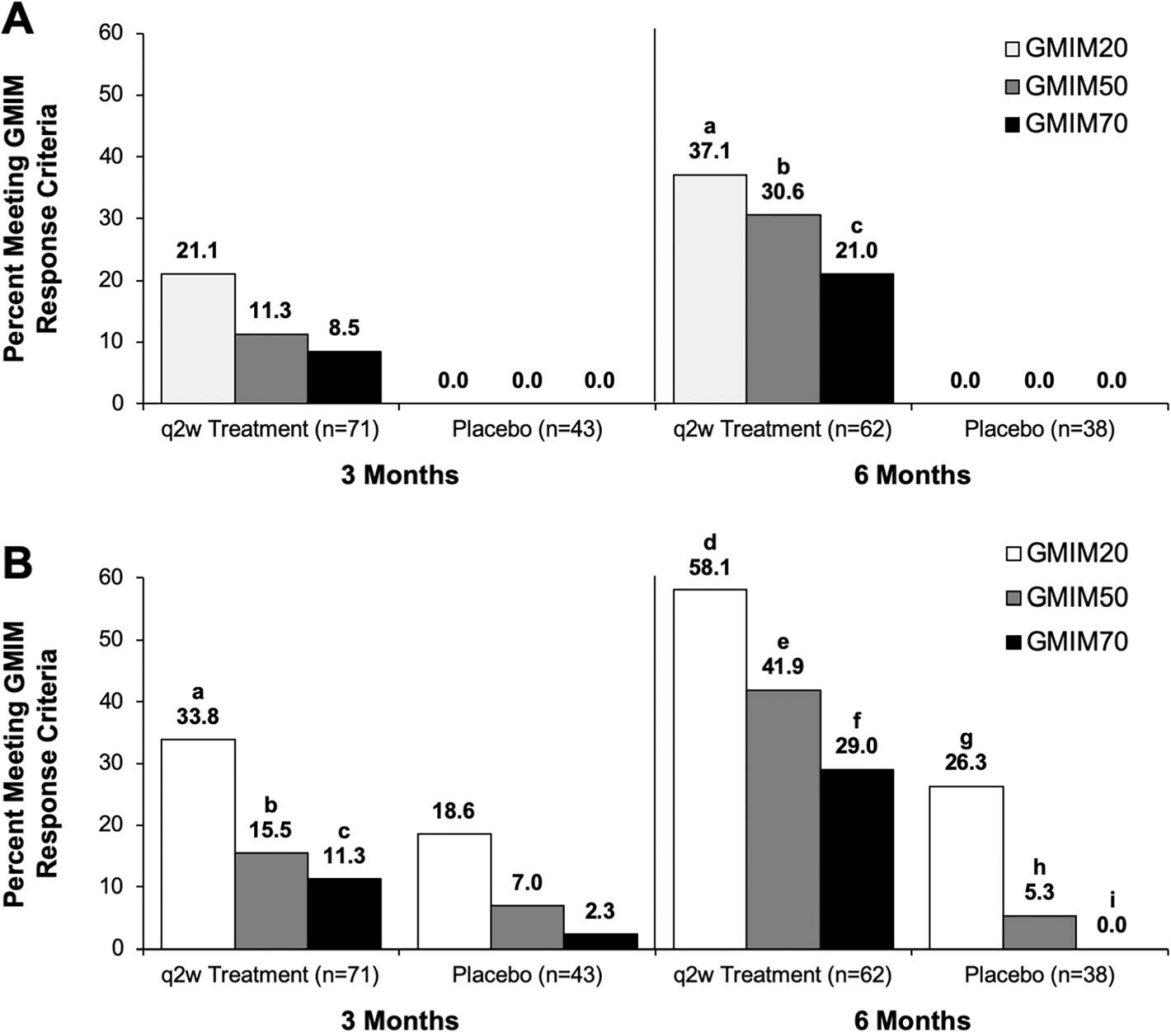
Percentage of subjects treated with pegloticase q2w or placebo reaching GMIM criteria at 3 and 6 months in the RCTs. Number of evaluable subjects is shown for each group. The percentage of responders is shown above the bars. **a** Results for all GMIM criteria and **b** Results for all criteria except urate < 6.0 mg/dL. The statistical analysis of the data is shown in the supplementary material

GMIM components (i.e., sU < 6 mg/dL, flares, tophi, PGA, pain, and swollen and tender joints) were determined from previous analyses of the patient data in the RCTs of pegloticase. A combination of criteria from the proposed remission criteria [33] and also criteria for a complete response [34] were considered. Although some criteria contributed little to the complete response model, such as pain and flares [34], they were included in the final GMIM because they were thought to be important in evaluating patients with gout. Moreover, it was felt that the way the data had been collected in the RCTs may have decreased its discriminative value [34]. For example, pain was collected as a general condition and not as related to gout. The previous exercise had evaluated the criteria strictly as a means to evaluate the induction of remission or complete response [34]. Here, we employed these criteria to determine the degree of response. Initially, the entire group of 85 subjects receiving biweekly pegloticase was assessed for the frequency of subjects achieving GMIM20, 50, and 70 status and compared to the outcome of subjects receiving placebo (Fig. 1a, supplementary table 1). No placebo-treated subjects achieved GMIM status, whereas approximately 40% of pegloticase-treated subjects became GMIM20 responders after the 6-month RCT. Since some pegloticase-treated subjects only had a transient urate-lowering effect, we were interested in determining whether pegloticase might have a clinical benefit even if the contemporaneous sU was not < 6 mg/dL. To accomplish this, we relaxed the GMIM status by omitting the requirement for a sU < 6 mg/dL. As can be seen in Fig. 1b and supplementary table 1, the frequency of GMIM responders was considerably higher in the pegloticase-treated subjects when the urate lowering requirement was removed, with approximately 60% of pegloticase-treated subjects achieving a GMIM20 and 30% a GMIM70 after 6 months of treatment. However, in this circumstance, nearly 30% of placebo-treated subjects also achieved a GMIM20 by 6 months, but only infrequently achieved a higher level of response.

**Fig. 2:**
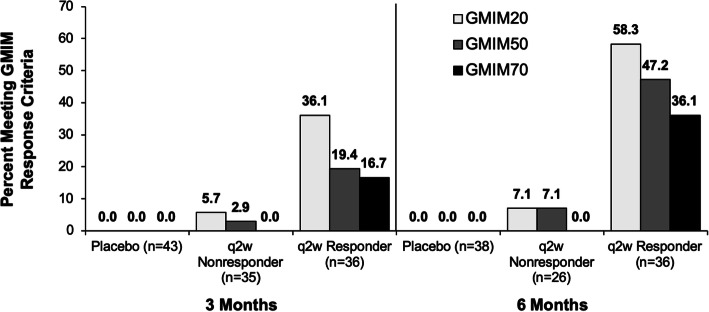
Percentage of responders and nonresponders to pegloticase or placebo-treated subjects meeting GMIM criteria at 3 and 6 months. Data show percentage of subjects meeting GMIM criteria including serum urate < 6 mg/dL. Number of evaluable subjects is shown for each group. The percentage of responders is shown above the bars. The statistical analysis of the data is shown in the supplementary material

To explore the relationship between persistent urate lowering caused by pegloticase and the percentage of subjects achieving GMIM responses in greater detail, we divided the pegloticase-treated subjects into those with persistent urate lowering (responders) and those with only transient urate lowering (nonresponders) and analyzed the percentage achieving GMIM responses. Thirty-four of 85 subjects treated with biweekly pegloticase had persistent urate lowering (responders) and also entered the OLE. As can be seen in Fig. 2 and supplementary table 2, GMIM responder status was mainly limited to the subjects with persistent urate lowering, with 36.1% and 58.3% achieving GMIM20 responses by 3 and 6 months, respectively. Less than 10% of subjects who failed to maintain persistent urate lowering (nonresponders) attained GMIM20 responses. It should be noted that many of these nonresponders still had urate lowering through 3 months of therapy and a small number had urate lowering at 6 months even though they did not meet the strict criteria of being a responder [34].

**Fig. 3:**
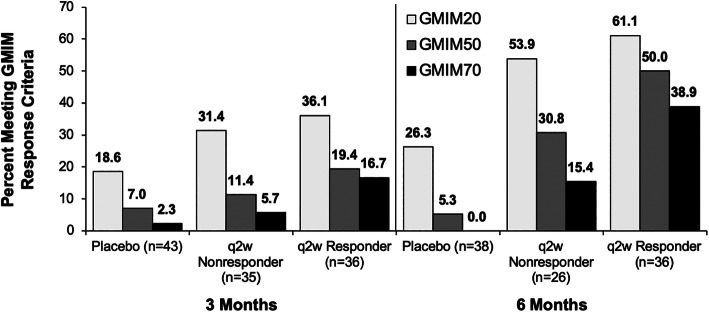
Percentage of responders and nonresponders to pegloticase or placebo-treated subjects meeting GMIM criteria at 3 and 6 months. Data show percentage of subjects meeting GMIM criteria excluding serum urate < 6 mg/dL. Number of evaluable subjects is shown for each group. The percentage of responders is shown above the bars. The statistical analysis of the data is shown in the supplementary material

To explore the clinical response in the nonresponders in greater detail, the criterion for achievement of sU < 6 mg/dL was omitted (Fig. 3, supplementary table 3). As with the analysis shown in Fig. 1, this resulted in some subjects in the placebo group achieving GMIM20, 50, and 70 responses at 3 months and GMIM20 and 50 responses at 6 months. There were also increases in GMIM20, 50, and 70 responses at 3 and 6 months for the q2w nonresponders. At 6 months, q2w responders achieved GMIM20 significantly more often vs placebo or nonresponders, whereas the nonresponders exhibited more frequent responses compared to placebo-treated subjects.

### Sensitivity analysis

**Fig. 4:**
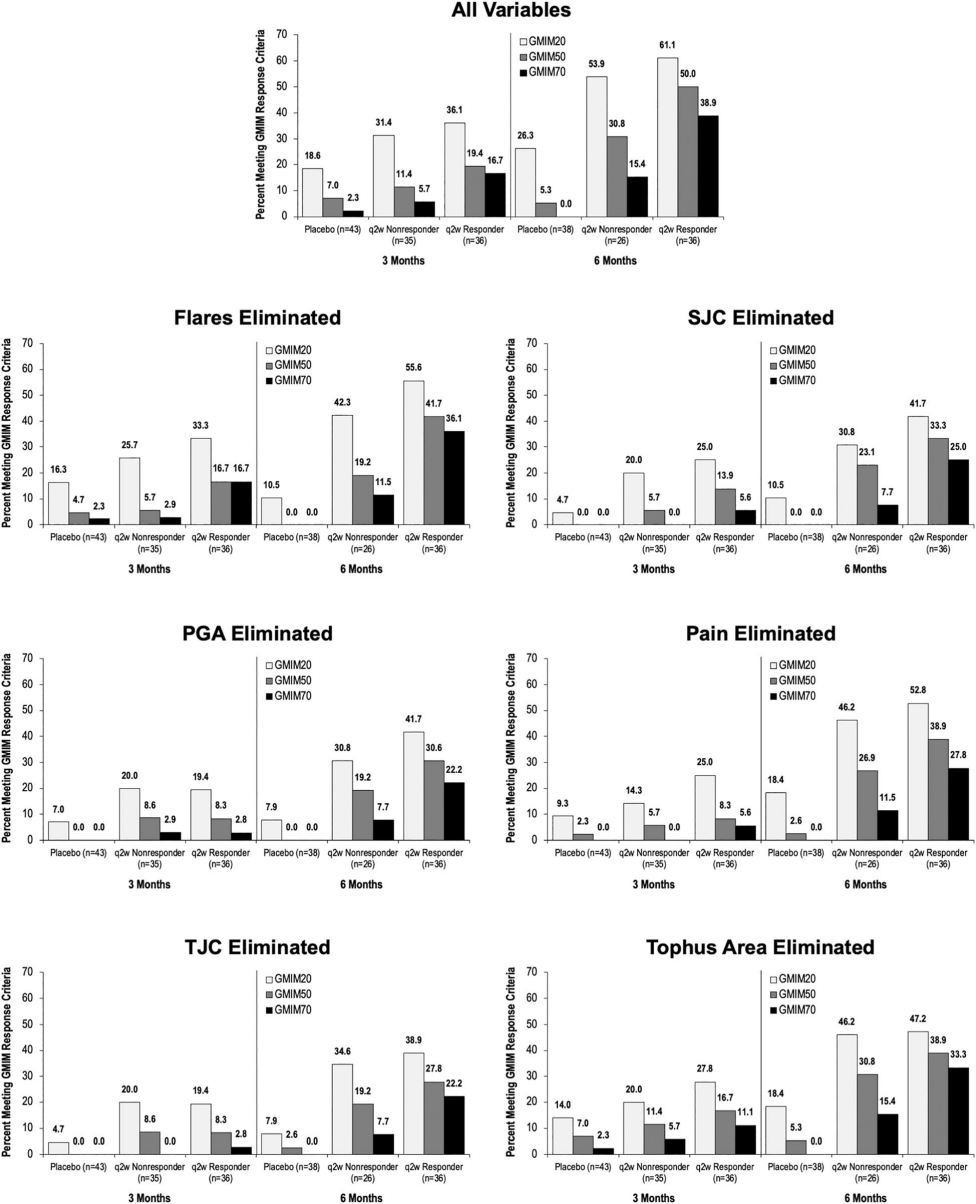
Sensitivity analysis of the GMIM model. Data show the percentage of responders and nonresponders to pegloticase or placebo-treated subjects meeting GMIM criteria at 3 and 6 months. Data show percentage of subjects meeting the GMIM criteria excluding serum urate < 6 mg/dL. In each figure, data are calculated omitting one criterion. Number of evaluable subjects is shown for each group. The percentage of responders is shown above the bars. Data are shown in supplementary table 4

A sensitivity analysis was carried out by recalculating GMIM20, 50, and 70 for each group with elimination (one at a time) of each of the clinical parameters. The requirement to achieve sU < 6 mg/dL was also not required for these analyses (Fig. 4, supplementary table 4). As can be seen, most variables contributed to the model with the exception of flares, whose elimination had little impact on the percentage of subjects meeting GMIM criteria. To explore this further, we carried out regression analysis of all of the GMIM components. Multiple linear regression analysis indicated that the components that consistently contributed information to GMIM were sU, PGA, tender joints, pain, and swollen joints. Tophus area and flare contributed only inconsistently. Because only a subset of patients had measurable tophi, we evaluated the performance of GMIM in those subjects independently (Supplementary figure 1). GMIM effectively separated responses in the tophaceous patients. Moreover, eliminating resolution of tophus area as a component of GMIM significantly decreased the measured responses, implying that tophus resolution significantly contributed to the GMIM model when only those subjects with tophi were considered.

### Achievement and maintenance of GMIM responses

**Fig. 5:**
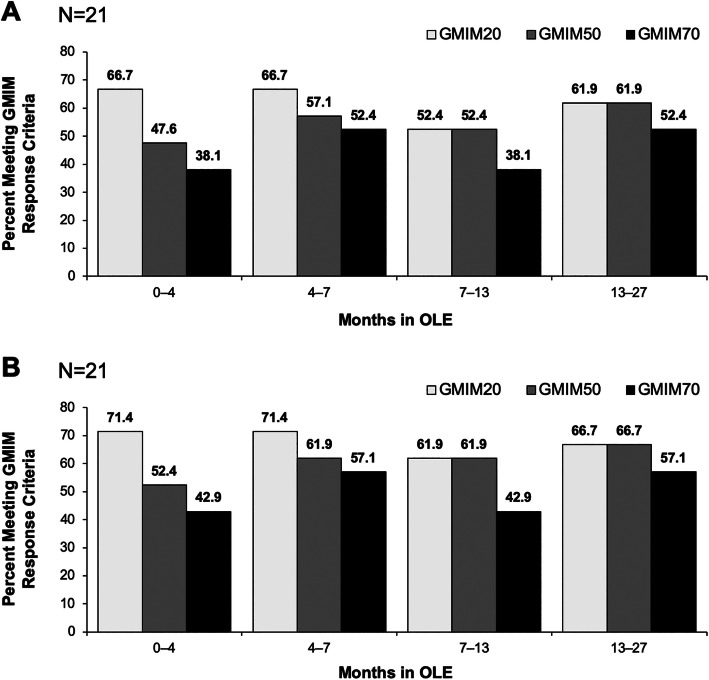
Percentage of subjects with GMIM20, 50, and 70 responses during the OLE for patients treated with biweekly pegloticase in the RCT and OLE. **a** Results for all criteria including urate < 6.0 mg/dL and **b** Results for all GMIM criteria excluding urate < 6 mg/dL

**Fig. 6:**
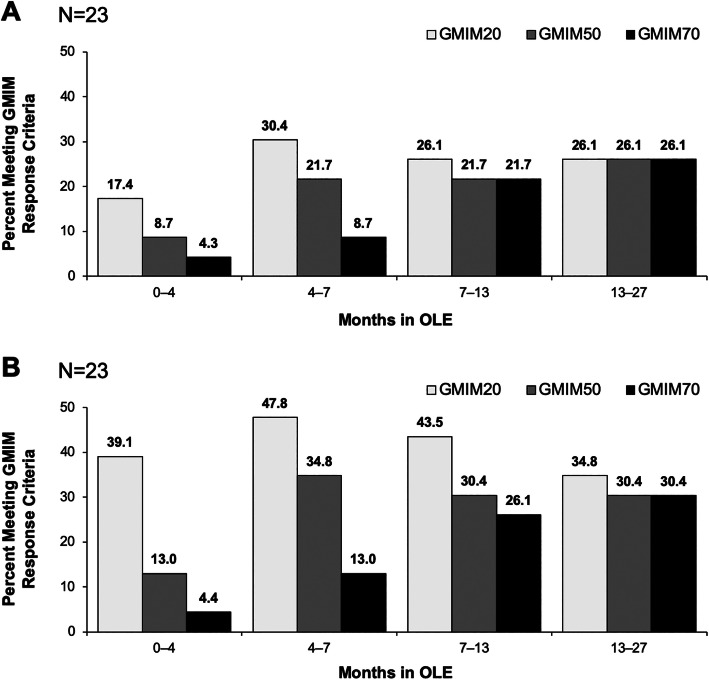
Percentage of subjects with GMIM20, 50, and 70 responses during the OLE for patients who were treated with placebo in the RCT and biweekly pegloticase in the OLE. **a** Results for all GMIM criteria including urate < 6 mg/dL and **b** Results for all criteria excluding urate < 6.0 mg/dL

Subjects who had persistent lowering of urate during the RCT were followed into the OLE. As can be seen, GMIM responses were maintained for the 2 years of the OLE (Fig. 5a, b). Moreover, patients who received placebo in the RCTs and were then switched to pegloticase q2w in the OLE achieved GMIM responses comparable to those treated with biweekly pegloticase in the RCTs (Fig. 6a, b).

## Discussion

The results from this analysis indicate that the GMIM criteria are useful as a composite outcome measure to capture response to pegloticase in chronic refractory gout patients. The measures comprising the composite outcome measure included sU, tophus area, SJC, TJC, PGA, flares, and pain. The first five were based on a repeated measures mixed effects model with backward elimination [34] using data from the pegloticase RCTs; pain was added given the importance attributed to it by patients with gout [36, 37] and the fact that it was a component of the original remission criteria [33]. Inclusion of this endpoint into the composite GMIM is consistent with results from studies that have evaluated the importance of different symptoms in patients with acute or chronic gout [16, 30]. Results from interviews of 30 patients with gout (10 with and 20 without clinically apparent tophi) indicated that pain was identified as being the cardinal, defining symptom of gout, leading to a range of impacts on health-related quality of life, most notably physical functioning and sleep [16]. Flares are included in the recommendations for elements in composite measures for assessment of gout treatment [28], the GAS composite [31], and proposed remission criteria [33], and this prompted their inclusion in the GMIM composite.

Results from the sensitivity analysis carried out in the present study showed that flares did not contribute significantly to the composite and that the contribution of pain was modest. This is not to suggest that flares are not important to patients, but just that they did not add information to the GMIM model. This could relate to the way information was collected in the RCTs. Flares were self-reported and pain was general and non-specifically related to gout. More precise collection of these variables could add important information to a multivariable outcome model.

Evaluation of results eliminating the requirement for sU < 6 mg/dL increased the percentages of patients achieving GMIM20, 50, and 70 among the q2w subjects without persistent urate lowering and also resulted in small numbers of placebo-treated patients achieving these goals. The observation that the percentages of subjects without persistent urate lowering achieving GMIM20, 50, and 70 at 6 months is consistent with previous analyses of results from these studies indicating significant clinical improvements in these patients despite failure to achieve sustained urate lowering [38]. It has been suggested that these improvements may be related to the transient but profound reductions in urate observed in these patients [38]. Results from the OLE showed that GMIM responses were sustained in the patients who initiated pegloticase treatment in the RCT and continued in the OLE. They also demonstrated that switching from placebo to q2w pegloticase treatment when progressing from the RCTs to the OLE resulted in clinical improvement comparable to that observed for those who initially received pegloticase and responded with persistent urate lowering in the RCTs and continued on this dose in the OLE. The ability of patients to “catch up” after delayed initiation of pegloticase should not be surprising since the patients randomized to placebo in the RCTs had gout symptoms for a mean of ~ 13 years at the time of study entry [26]. Importantly, patients switching from placebo to pegloticase were not included in the generation of GMIM and, therefore, serve to validate the approach.

As noted above, a composite endpoint for assessment of gout treatment was developed before the GMIM. The GAS was based on longitudinal analysis of results from a multicenter observational cohort study (Kick-Off of the Italian Network for Gout [KING]), in which 68.7% of a cohort of 406 patients were treated with allopurinol and 13.6% were treated with febuxostat [31]. A multistep process that began with all measures in the OMERACT core domain [20] and included factor analysis, linear discriminant analysis, and linear regression resulted in inclusion of four factors: gout flares in the past 12 months, sU, pain, and number of tophi [31]. All of these measures were included in GMIM and both TJC and SJC were also added into GMIM, which differed from the GAS in that it was focused on responsivity to pegloticase treatment. Recently, Chinchilla et al. confirmed the predictive validity of the GAS, its correlation with the GIS, and its sensitivity to change [32]. It is important to note that the disease severity for patients enrolled in the KING study which provided the basis for the GAS was very different from those in the RCTs for pegloticase [26, 31]. The patients in the pegloticase studies had longer duration of disease (12–16 years across treatment groups vs 3.8 years), greater numbers of tender (11.1–14.1 vs 1) and swollen (8.9–13.2 vs 0) joints, tophi (65.2% to 76.7% vs 19.5%), flares (20–43 over 18 months before treatment vs 0 over 3 months), and sU (9.4–10.4 mg/dL vs 6.3 mg/dL). In addition, patients enrolled in the pegloticase RCTs were required to have gout that was refractory to allopurinol treatment or to be intolerant of this medication whereas this was not the case in the KING study. GMIM was, therefore, tested in a very severe group of subjects with advanced gout. Whether it would be effective in subjects with less advanced disease remains to be evaluated, but the overlapping components of GAS and GMIM suggest that it might. A parallel effort to identify a multivariable outcome measure for acute gout might be worthwhile. Moreover, a direct comparison of the performance of GAS and GMIM might be informative.

There is an important limitation with respect to the GMIM as it is currently formulated. There is no adjustment for baseline variables with scores so low that the patient cannot achieve a specified (20%, 50%, or 70%) improvement regardless of the efficacy of treatment. This same problem applies to the American College of Rheumatology criteria for assessment of treatments for rheumatoid arthritis [39, 40] and has been used as a justification for composite measures that assess absolute disease activity vs change from baseline [40]. This limitation might be overcome by adjusting response criteria to include reduction of a given endpoint to 0 when baseline scores are sufficiently low. We addressed this issue by making the response criterion for a response improvement, 3 of the 5 GMIM parameters in addition to achieving sU < 6 mg/dL and a decrease in flares. Finally, there is a general concern about composite criteria and whether they might overestimate responses has been articulated [41, 42] For this reason, GMIM was structured to have the 2 most frequently required components, decrease in sU < 6.0 mg/dL and decrease in flares to be mandatory components.

## Conclusion

In summary, the GMIM criteria effectively capture a change in disease severity in chronic refractory gout patients treated with pegloticase. It may serve as an evidence-based tool for assessment of therapies used to treat patients with similar baseline disease severity.

## Supplementary information

**Additional file 1.**

**Additional file 2.**

**Additional file 3.**

**Additional file 4.**

**Additional file 5.**

## Data Availability

The data and analytic methods that support the findings of this study are available to qualified investigators by request to the corresponding author.

## Abbreviations

GAS: Gout Activity Score
GIS: Gout Impact Scale
GMIM: Gout Multivariable Improvement Measure
HAQ: Health Assessment Questionnaire
KING: Kick-off of the Italian Network for Gout
OLE: Open-label extension
OMERACT: Outcome Measures in Rheumatology
PGA: Patient global assessment
RCT: Randomized controlled trial
SJC: Swollen joint count
sU: Serum urate
TJC: Tender joint count
ULT: Urate-lowering therapy
VAS: Visual analog scale
