# Supplementary figures and images for: Development of a multivariable improvement measure for gout
Source: Arthritis Res Ther. 2020 Jun 29;22:164. doi: 10.1186/s13075-020-02254-4 (PMC7325077; doi:10.1186/s13075-020-02254-4)

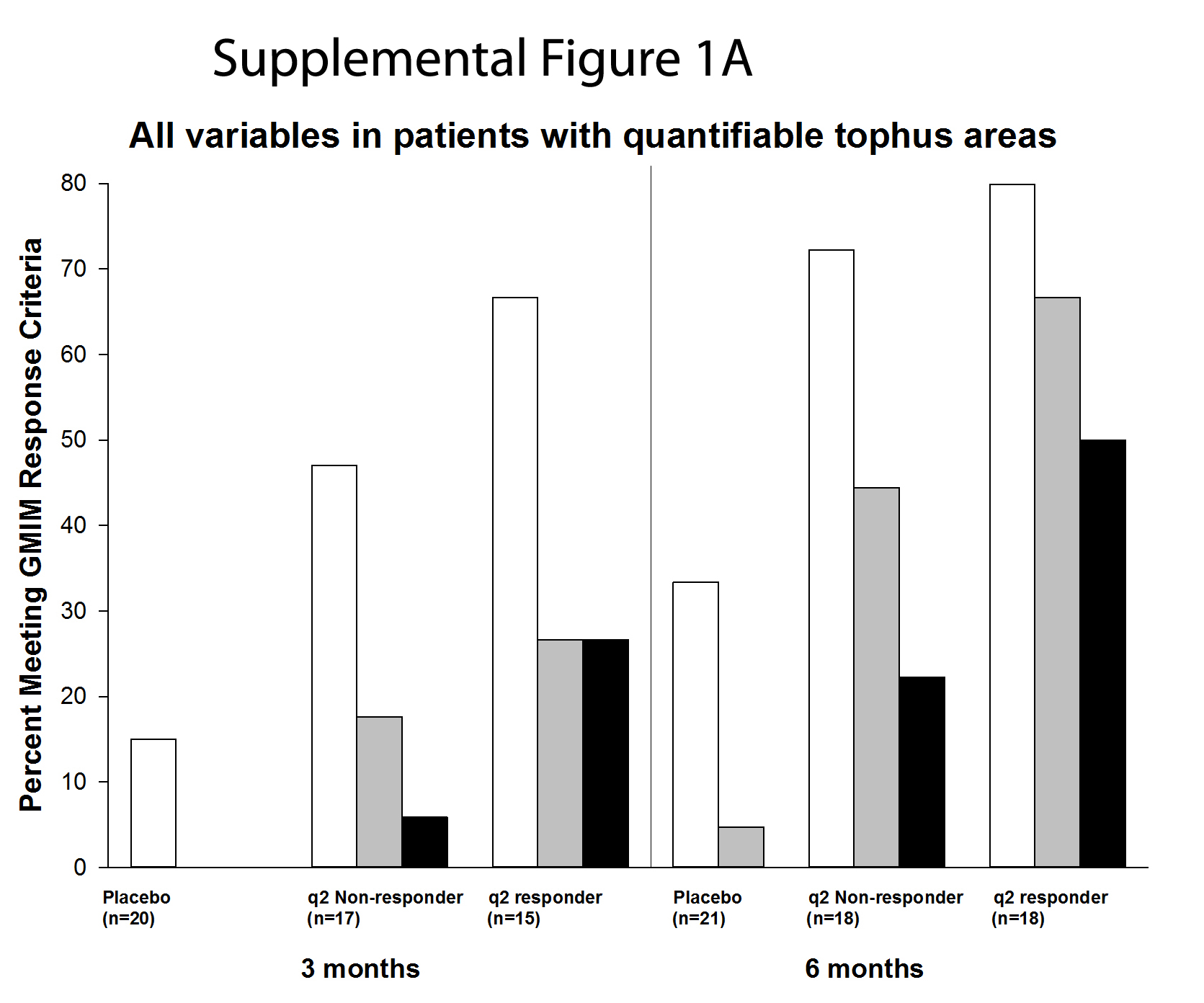

Supplement: Supplementary file 1 — Additional file 1. [file 13075_2020_2254_MOESM1_ESM.zip › Supplemental Figure 1A_Tophus_all_variable.jpg]

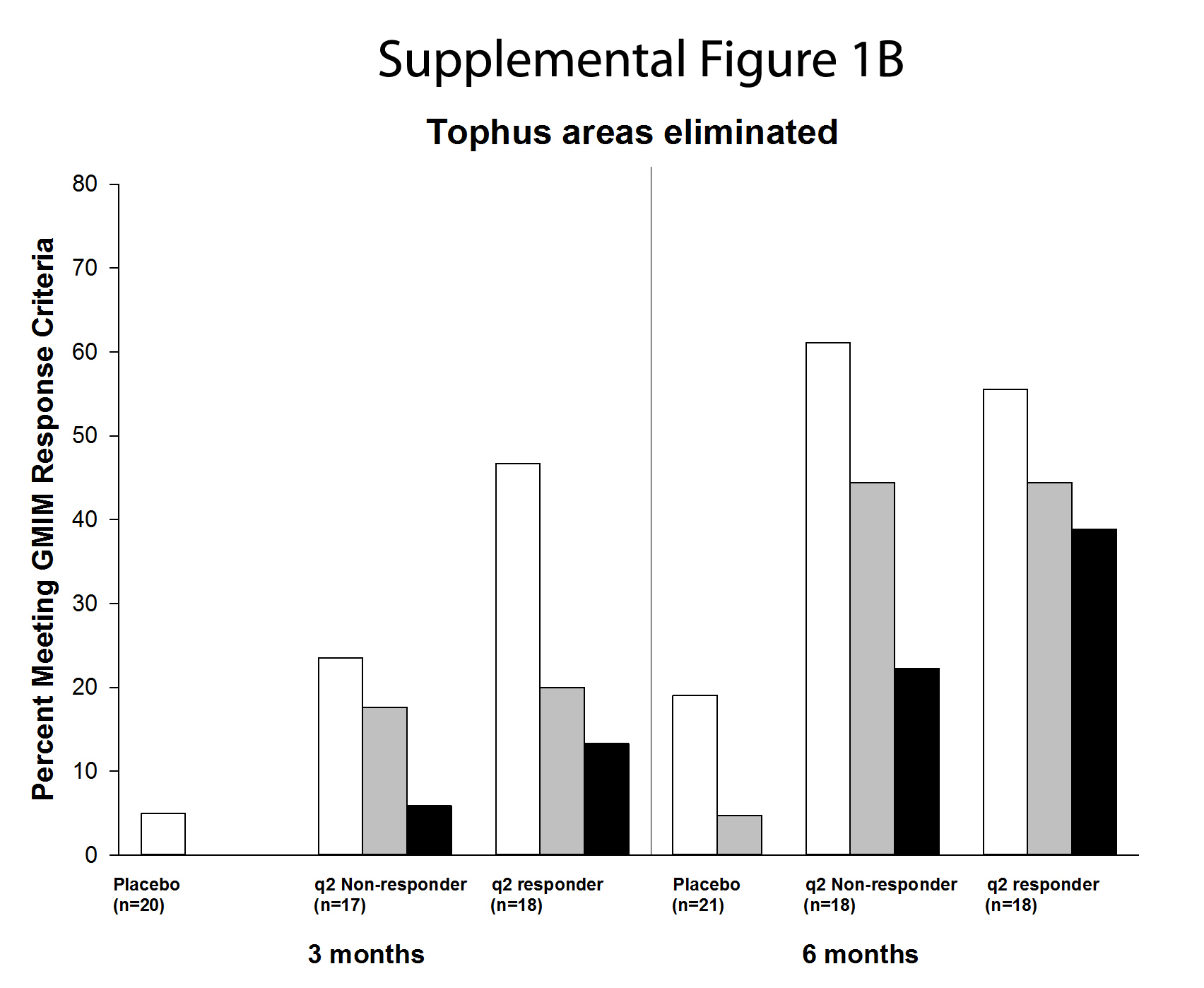

Supplement: Supplementary file 1 — Additional file 1. [file 13075_2020_2254_MOESM1_ESM.zip › Tophus_areas_eliminated2a-2.jpg]
