# Supplementary material for: Development of a multivariable improvement measure for gout
Source: Arthritis Res Ther. 2020 Jun 29;22:164. doi: 10.1186/s13075-020-02254-4 (PMC7325077; doi:10.1186/s13075-020-02254-4)
Supplement: Supplementary file 2 — Additional file 2. [file 13075_2020_2254_MOESM2_ESM.docx]

Supplementary Table 1. Statistical analysis of data shown in Figure 1

| **Figure 1A**  Pegloticase vs placebo | **GMIM 20** | **GMIM 50** | **GMIM 70** |
| --- | --- | --- | --- |
| 3 months | 0.0009 | 0.024 | 0.082 |
| 6 months | <0.0001 | <0.0001 | 0.0015 |
| Pegloticase 3months vs 6 months | 0.086* | 0.014* | 0.070* |
| **Figure 1B**  Pegloticase vs placebo | **GMIM 20** | **GMIM 50** | **GMIM 70** |
| 3 months | 0.12* | 0.24* | 0.15* |
| 6 months | 0.0039* | <0.0001 | <0.0001 |
| Pegloticase 3 months vs 6 months | 0.0085* | 0.0014* | 0.018* |

All values calculated by Fisher’s exact test except those designated by * calculated by continuity adjusted Chi square
