# Supplementary material for: Development of a multivariable improvement measure for gout
Source: Arthritis Res Ther. 2020 Jun 29;22:164. doi: 10.1186/s13075-020-02254-4 (PMC7325077; doi:10.1186/s13075-020-02254-4)
Supplement: Supplementary file 3 — Additional file 3. [file 13075_2020_2254_MOESM3_ESM.docx]

Supplementary Table 2. Statistical analysis of the data shown in Figure 2

| **Pegloticase vs placebo** | **GMIM 20** | **GMIM 50** | **GMIM 70** |
| --- | --- | --- | --- |
| 3 months – Non-responders | 0.20 | 0.45 | - |
| 3 months - Responders | <0.0001 | 0.0029 | 0.007 |
| 6 months – Non-responders | 0.18 | 0.18 | - |
| 6 months – Responders | <0.0001 | <0.0001 | <0.0001 |
| **Pegloticase**  **3 months vs 6 months** | **GMIM 20** | **GMIM 50** | **GMIM 70** |
| Non-responders | 1.0 | 0.58 | - |
| Responders | 0.10 | 0.024 | 0.1 |

All p-values calculated by Fisher’s exact test.
