# Supplementary material for: Development of a multivariable improvement measure for gout
Source: Arthritis Res Ther. 2020 Jun 29;22:164. doi: 10.1186/s13075-020-02254-4 (PMC7325077; doi:10.1186/s13075-020-02254-4)
Supplement: Supplementary file 4 — Additional file 4. [file 13075_2020_2254_MOESM4_ESM.docx]

Supplementary Table 3. Statistical analysis of data shown in Figure 3

Significance of Differences

| **Pegloticase vs placebo** | **GMIM 20** | **GMIM 50** | **GMIM 70** |
| --- | --- | --- | --- |
| 3 months – Non-responders | 0.30* | 0.69 | 0.58 |
| 3 months – Responders | 0.13* | 0.17* | 0.04* |
| 6 months – Non-responders | 0.049* | 0.011 | 0.024 |
| 6 months – Responders | 0.054* | <0.0001 | <0.0001 |
| **Pegloticase**  **3 months vs 6 months** | **GMIM 20** | **GMIM 50** | **GMIM 70** |
| Non-responders | 0.13* | 0.10 | 0.39 |
| Responders | 0.059* | 0.01* | 0.66* |

All p-values calculated by Fisher’s exact test except those designated by a * calculated by continuity adjusted Chi square
