# Supplementary material for: Development of a multivariable improvement measure for gout
Source: Arthritis Res Ther. 2020 Jun 29;22:164. doi: 10.1186/s13075-020-02254-4 (PMC7325077; doi:10.1186/s13075-020-02254-4)
Supplement: Supplementary file 5 — Additional file 5. [file 13075_2020_2254_MOESM5_ESM.docx]

Supplementary Table 4.

Significance of Differences

| **Percent Achieving** | **GMIM 20**  **%** | **GMIM 50**  **%** | **GMIM 70**  **%** |
| --- | --- | --- | --- |
| **All Variables** | | | |
| 3 months – Placebo | 18.6 | 7.0 | 2.3 |
| 3 months – Nonresponders | 31.4 | 11.4 | 5.7 |
| 3 months – Responders | 36.1 | 19.4 | 16.7 |
| 6 months – Placebo | 26.6 | 5.3 | 0 |
| 6 months – Nonresponders | 53.9 | 30.8 | 15.4 |
| 6 months – Responders | 61.1 | 50.0 | 38.9 |
| **Flares Eliminated** | | | |
| 3 months – Placebo | 16.3 | 4.7 | 2.3 |
| 3 months – Nonresponders | 25.7 | 5.7 | 16.7 |
| 3 months – Responders | 33.3 | 16.7 | 16.7 |
| 6 months – Placebo | 10.5 | 0 | 0 |
| 6 months – Nonresponders | 42.3 | 19.2 | 11.5 |
| 6 months – Responders | 55.6 | 41.7 | 36.1 |
| **SJC Eliminated** | | | |
| 3 months – Placebo | 4.7 | 0 | 0 |
| 3 months – Nonresponders | 20.0 | 5.7 | 0 |
| 3 months – Responders | 25.0 | 13.9 | 5.4 |
| 6 months – Placebo | 10.5 | 0 | 0 |
| 6 months – Nonresponders | 30.8 | 21.1 | 7.7 |
| 6 months – Responders | 41.7 | 33.3 | 25.0 |
| **PGA Eliminated** | | | |
| 3 months – Placebo | 7.0 | 0 | 0 |
| 3 months – Nonresponders | 20.0 | 8.6 | 2.9 |
| 3 months – Responders | 19.4 | 8.3 | 2.8 |
| 6 months – Placebo | 7.9 | 0 | 0 |
| 6 months – Nonresponders | 30.8 | 19.2 | 7.7 |
| 6 months – Responders | 41.7 | 30.6 | 22.2 |
| **Pain Eliminated** | | | |
| 3 months – Placebo | 9.3 | 2.3 | 0 |
| 3 months – Nonresponders | 14.3 | 5.7 | 0 |
| 3 months – Responders | 25.0 | 8.3 | 5.6 |
| 6 months – Placebo | 18.4 | 2.6 | 0 |
| 6 months – Nonresponders | 46.2 | 26.9 | 11.5 |
| 6 months – Responders | 52.8 | 38.9 | 27.8 |
| **TJC Eliminated** | | | |
| 3 months – Placebo | 4.7 | 0 | 0 |
| 3 months – Nonresponders | 20.0 | 8.6 | 0 |
| 3 months – Responders | 19.4 | 8.3 | 2.8 |
| 6 months – Placebo | 7.9 | 2.6 | 0 |
| 6 months – Nonresponders | 34.6 | 19.2 | 7.7 |
| 6 months – Responders | 38.9 | 27.8 | 22.2 |
| **Tophus Area Eliminated** | | | |
| 3 months – Placebo | 14.0 | 7.0 | 2.3 |
| 3 months – Nonresponders | 20.0 | 11.4 | 5.7 |
| 3 months – Responders | 27.8 | 16.7 | 11.1 |
| 6 months – Placebo | 18.4 | 5.3 | 0 |
| 6 months – Nonresponders | 46.2 | 30.8 | 15.4 |
| 6 months – Responders | 47.2 | 38.9 | 33.3 |
